# Supplementary material for: Habitat quality, configuration and context effects on roe deer fecundity across a forested landscape mosaic
Source: PLoS One. 2019 Dec 27;14(12):e0226666. doi: 10.1371/journal.pone.0226666 (PMC6934308; doi:10.1371/journal.pone.0226666)
Supplement: S1 File — (DOCX) [file pone.0226666.s001.docx]

**S1 File. Study landscape.**

Thetford Forest is a pine-dominated plantation landscape; during the study period it comprised Corsican, *Pinus nigra* (55% of the landscape), and Scots *Pinus sylvatica* (17%) pines, *Larix* spp., open habitats including clearfelled areas (18%) and some mature deciduous plantings (particularly *Fagus sylvatica*, *Acer pseudoplatanus* and *Betula* spp.) (together 10%). Most of the forest (62% of planted crops) was in the second crop rotation.

For spatial analysis, the twelve forest ‘blocks’ recognised as management units by the Forestry Commission (that differed greatly in size and included heterogeneous blocks) were further divided into 14 ‘subregions’ (mean area= 13.2 km^2^, SD= 5.6), that differed in landuse character, particularly the representation of soil types and configuration. These subregions were included as *a priori* random effects in roe deer fecundity and body mass models in order to account for unmeasured variance between locations. A number of conifer-dominated subregions on primarily acidic soils (podsols and gravelly sands) formed an extensive contiguous core, while smaller outlying subregions on more diverse soils (including sandy calcareous rendzinas or limited areas of wetter gleys or peats) had greater perimeter (defined as subregion boundary adjoining arable or grasslands) to area ratio and greater tree crop diversity. Extent of calcareous soil, subregion area, perimeter, and perimeter-area ratio metrics of these 14 subregions were independent, while having some replication of multiple subregions within each class (Table 1). Unsupervised cluster analysis (hierarchical clustering and average agglomeration method in R (1) using “stats” package ) of perimeter-area ratio and percentage of calcareous soil resolved one group comprising four larger subregions (Kings, Mundford, Elveden and High Lodge), two of which are more calcareous while the other two are more acidic); and three groups of smaller subregions; of which two had greater perimeter-area ratio (one more calcareous group: Harling, Swaffham, Didlington and Hockwold; one more acidic group: Croxton, Mildenhall, Hockham) and one comprised subregions with moderate or low perimeter-area ratio (Santon Downham, West Tofts, Lynford) (Fig 1). These groupings of subregions confirm independent replicates (located in different parts of the forested landscape) for each combination of variables.

**Table 1: Thetford forest’s subregions.** Subregions of Thetford Forest, showing area, perimeter, perimeter area ratio and percentage of calcareous soil. Shading indicates deviation of the subregion-specific measure from the mean value (as % of the mean: 0-25%, 26-50%, 51-75%, 76-100%, >100%), red positive (> mean) green negative (< mean), lighter shades indicated smaller deviation while darker indicated larger deviation.

| **Cluster ID** | **Subregion number** | **Subregion name** | **Area (km^2^)** | **Perimeter (km)** | **Perimeter-Area ratio** | **Percentage calcareous soil** |
| --- | --- | --- | --- | --- | --- | --- |
| 1 | 5 | Lynford | 11.8 | 6.1 | 0.5 | 30.4 |
|  | 6 | West Tofts | 8.9 | 13.8 | 1.5 | 29.7 |
|  | 10 | Santon  Downham | 11.9 | 1.2 | 0.1 | 34.8 |
| 2 | 11 | High Lodge | 22.7 | 8.4 | 0.3 | 24.8 |
|  | 12 | Elveden | 16.5 | 6.0 | 0.3 | 18.9 |
|  | 13 | Kings | 23.3 | 26.0 | 1.1 | 42.5 |
|  | 4 | Mundford | 21.2 | 32.1 | 1.5 | 45.7 |
| 3 | 7 | Croxton | 10.2 | 26.2 | 2.7 | 19.9 |
|  | 8 | Hockham | 8.3 | 28.7 | 3.4 | 4.5 |
|  | 14 | Mildenhall | 5.9 | 15.7 | 2.6 | 15.9 |
| 4 | 1 | Swaffham | 13.3 | 50.9 | 3.8 | 42.8 |
|  | 2 | Didlington | 9.1 | 42.0 | 4.5 | 43.3 |
|  | 3 | Hockwold | 12.1 | 34.7 | 2.8 | 64.7 |
|  | 9 | Harling | 11.4 | 35.1 | 3.0 | 45.8 |
|  |  | **Mean** | 13.3 | 23.3 | 2.0 | 33.1 |

**
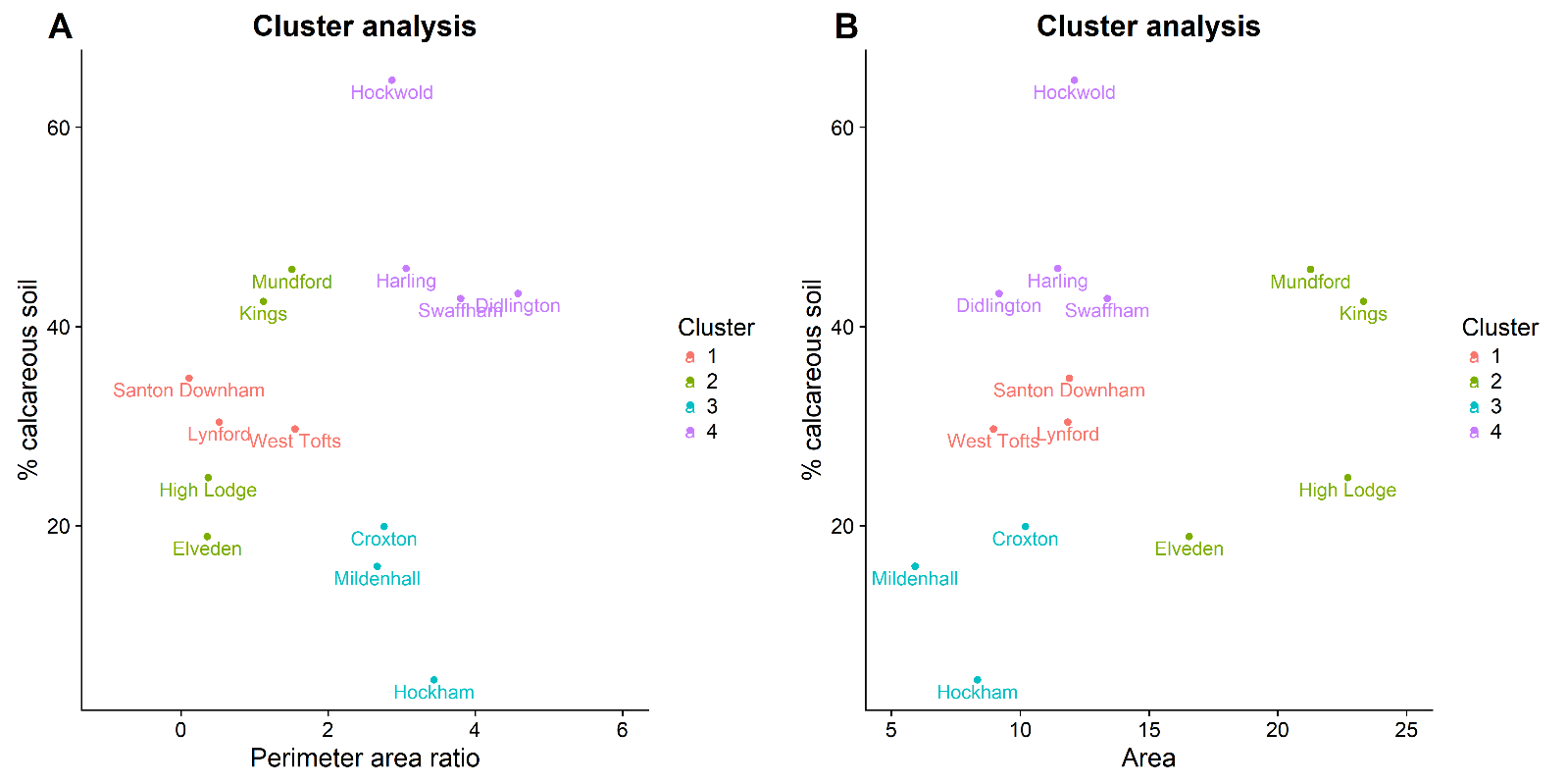
**

**Fig 1: Cluster analysis.** Cluster analysis, using hierarchical clustering and average agglomeration, of Thetford Forest subregions. The same colour was applied to forest subregions belonging to the same cluster. **References**

1. R core Team. R: A Language and Environment for Statistical Computing [Internet]. Vienna: R Foundation for Statistical Computing; 2018. Available from: https://www.r-project.org/
