# Supplementary material for: Habitat quality, configuration and context effects on roe deer fecundity across a forested landscape mosaic
Source: PLoS One. 2019 Dec 27;14(12):e0226666. doi: 10.1371/journal.pone.0226666 (PMC6934308; doi:10.1371/journal.pone.0226666)
Supplement: S2 File — (DOCX) [file pone.0226666.s002.docx]

**S2 File. Validation of the larder dataset**

Carcass damage was recorded in the Forestry Commission data for 5.4 % of adults and 5.5 % of yearlings. Carcass damage is reported, together with other additional information, in a “remark” text field. However, some culled animals were not recorded as damaged although their recorded body mass was implausibly low (relative to expert opinion); we consider this likely to represent occasional errors in data entry, which those responsible for data capture report is more likely to result from failure to record the damage code rather than error in entry of body mass. We therefore imposed a threshold of 6 kg for a minimum yearling’s carcass body mass and 8 kg for an adult; further excluding 1% of yearling and 2% of adult individuals not coded as damaged.

We excluded all data for animals culled by non-FC rangers active in one forest subregion (Mundford) as we considered those data were not recorded accurately while we retained the data collected from FC rangers from the same forest subregion.
