# Supplementary material for: Habitat quality, configuration and context effects on roe deer fecundity across a forested landscape mosaic
Source: PLoS One. 2019 Dec 27;14(12):e0226666. doi: 10.1371/journal.pone.0226666 (PMC6934308; doi:10.1371/journal.pone.0226666)
Supplement: S3 File — (DOCX) [file pone.0226666.s003.docx]

**S3 File. Effect of date on detectability of embryos**


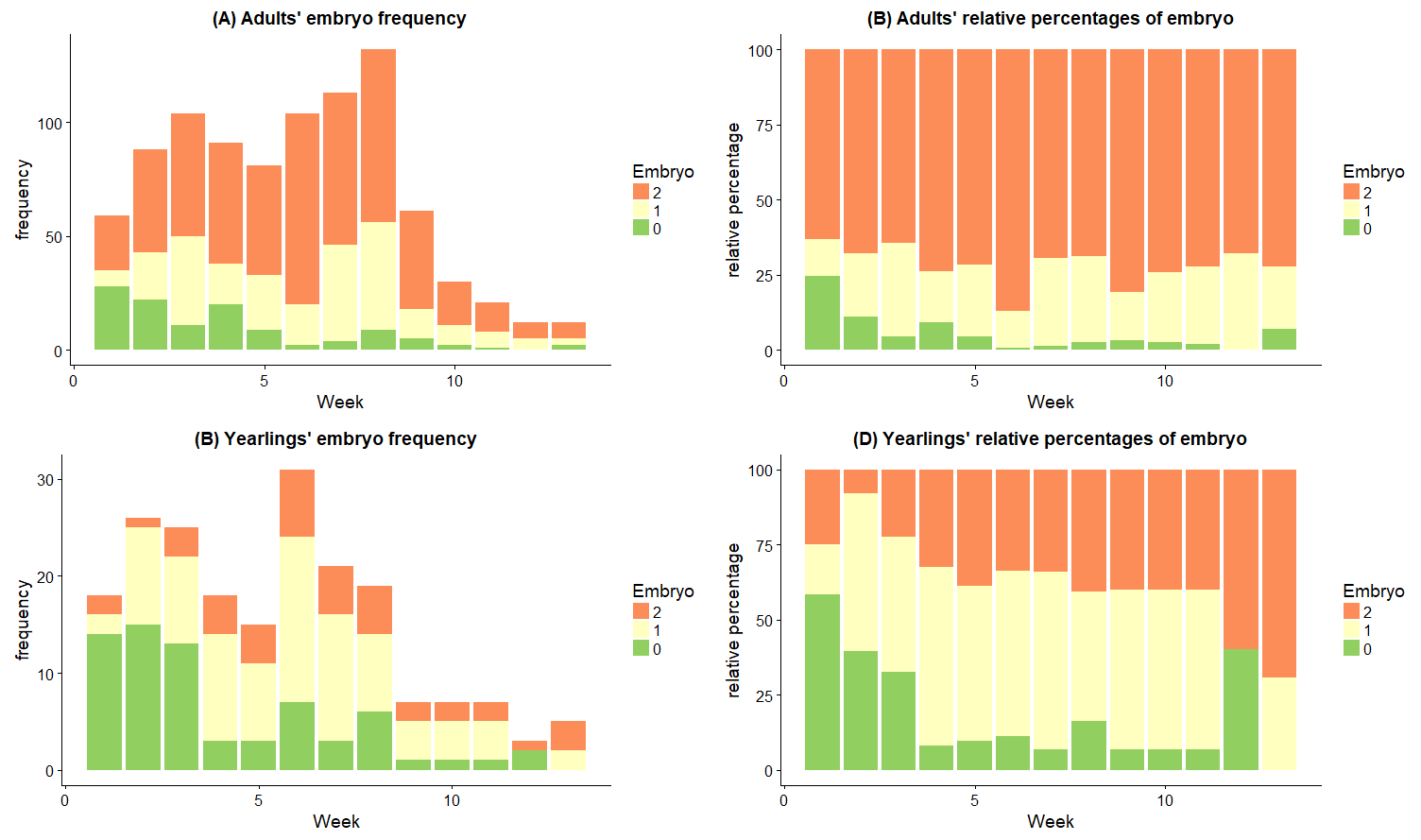
 **Fig 1**: Apparent yearling and adult roe deer fecundity in relation to calendar week, showing total frequency (per calender week) and relative percentage (as % of the total for that calender week) of uterine records with no, one or two embryos. Weeks span from 01 of January to the last week of March.

We explored univariate models relating number of embryos to calendar week, coded as: continuous; continuous square rooted; categorical with one factor for each week; or as dummy categorical variables coded either with: 0=week 1, 1=weeks 2-12; or 0=weeks 1-2, 1=weeks 3-12; or 0=week 1-3, 1=week 4-12. The most parsimonious model was determined by comparing goodness of fit (model AIC) and model complexity (degrees of freedom). For adults the best-fitting model used 0=weeks 1-3, 1=weeks 4-12; for yearlings the best-fitting models used 0=weeks 1-2, 1=weeks 3-12.

**Table 1**: Model selection of week coding for adults and yearlings fecundity. For each model the difference in AIC value relative to the best supported model (∆AIC) and the degrees of freedom (df) are shown.

|  | **Adults** | | | |
| --- | --- | --- | --- | --- |
| **Variable** | | **Variable df** | **∆AIC** | **df** |
| Week dummy (0=weeks 1-3, 1=weeks 4-12) | | 1 | 0 | 906 |
| Week dummy (0=weeks 1-2, 1=weeks 3-12) | | 1 | 4.3 | 906 |
| Week square rooted | | 1 | 4.9 | 906 |
| Week continuous | | 1 | 5.8 | 906 |
| Week dummy (0=week 1, 1=weeks 2-12) | | 1 | 6.5 | 906 |
| Week categorical | | 12 | -1.6 | 895 |
|  | **Yearlings** | | | |
| **Variable** | | **Variable df** | **∆AIC** | **df** |
| Week dummy (0=weeks 1-2, 1=weeks 3-12) | | 1 | 0 | 906 |
| Week dummy (0=weeks 1-3, 1=weeks 4-12) | | 1 | 1.2 | 906 |
| Week square rooted | | 1 | 3 | 906 |
| Week continuous | | 1 | 4 | 906 |
| Week dummy (0=week 1, 1=weeks 2-12) | | 1 | 6.2 | 906 |
| Week categorical | | 12 | 8.4 | 895 |
