# Supplementary material for: Habitat quality, configuration and context effects on roe deer fecundity across a forested landscape mosaic
Source: PLoS One. 2019 Dec 27;14(12):e0226666. doi: 10.1371/journal.pone.0226666 (PMC6934308; doi:10.1371/journal.pone.0226666)
Supplement: S4 File — (DOCX) [file pone.0226666.s004.docx]

**S4 File. Land cover and soil data**

Surrounding landcover was obtained from the Land Cover Map 2007 (LCM 2007; (1)), that classifies 23 classes based on UK Biodiversity Action Plan (BAP) Broad Habitats (2) from composite summer-winter satellite images (pixel resolution 20-30 m), spatially referenced to the Ordnance Survey MasterMap digital topographic layer, with a minimum mapped resolution of 0.5 ha (smaller parcels, and linear features less than 20m, are dissolved into the surrounding landscape). Overall classification accuracy based on field validation is reported as 83% across all LCM 2007 classes, but is acknowledged to be poorer for some ecologically-similar land cover types (1). To explore reliability of CEH LCM 2007 data within the study area, areas were compared with a Google image cross-referenced by OS map data (Fig 1 A and B) for parts of the landscape well known to the researchers. This showed that improved grassland, different semi-natural grassland types, and mixed dwarf shrub and grassland, were not reliably separated, with frequent misclassification in LCM 2007 of seminatural grassland classes (either rough, neutral, or calcareous grassland, heather, and heather grassland) that were instead classified as improved grassland. Furthermore, within Breckland grass-heaths, calcareous grassland, acidophilous grassland and dwarf-shrub (heathland) assemblages can be intimately mixed over scales of a few meters (3). Consequently, all CEH grassland types were merged and soil type was considered separately.

Two soil data layers were available and data were reconciled to a common classification. First, higher resolution (18 soil series, and their complexes, mapped to a minimum spatial resolution of 180 m) for the Thetford Forest were available from the Breckland forest soil map (4). Second, for the wider landscape, lower resolution data from Cranfield National Soil Map of England and Wales ((5), NATMAP) classify nine series mapped at 1:250,000 scale. These classifications were simplified to provide consistent classes of: acidic (including acidic complexes) and calcareous (including mixed calcareous/acid complexes) (Table 1, Fig. 2). They were validated by overlaying Breckland forest soil map and NATMAP data for those areas of Thetford Forest for which both were available (Fig. 1). Areas of wet soil types (gleys, peats) that comprised only a small proportion of the forest (5%) were excluded.

Soil was examined in terms of the proportionate extent of calcareous soil within the aggregate area of forest and grassland lying within each home-range buffer.

**Table 1:** Soil classification used in the study showing corresponding series and complexes from the Breckland Forest soil map and Cranfield NATMAP.

| Aggregate classes | Breckland forest soil map | Cranfield NATMAP |
| --- | --- | --- |
| Acidic soils | Acidic soils: bJ, bU, Fr, Ro, sN, Wk, Wt, Wt/Wt1, Wt/Wt2, Wt2; Acidic complexes: bJ/Fr, bJ/Fr/Ro, bU/Fr, bU/Fr/Ro, bU/Wt, r/Hh/Lx/rO, Fr/Ro, Fr/Ro/sN/Wt, Fr/Wt, Ro/Wt2, sN/Wt, sN/Wt1 | 0511g (Deep well drained sandy soils)  0554b (Deep well drained sandy soils in places very acid with subsurface pan) |
| Calcareous soils | Calcareous soils: Cr, Mo, Mw, Mw\Nf; Mixed complexes: cR/Fr, cR/Wt/Wt1 , Mo/Wt , Mw/Nf/Wt , Mw/Wt , Mw/Wt/Wt1 , Mw/Wt1 , Mw/Wt2 , Nf/Wt | 0343f (Shallow well drained calcareous sandy and coarse loamy soils over chalk or chalk rubble)  0343g(Shallow well drained calcareous coarse loamy and sandy soils over chalk rubble)  0511e (Well drained calcareous coarse and fine loamy soils over chalk rubble)  0521(Well drained calcareous sandy soils) |


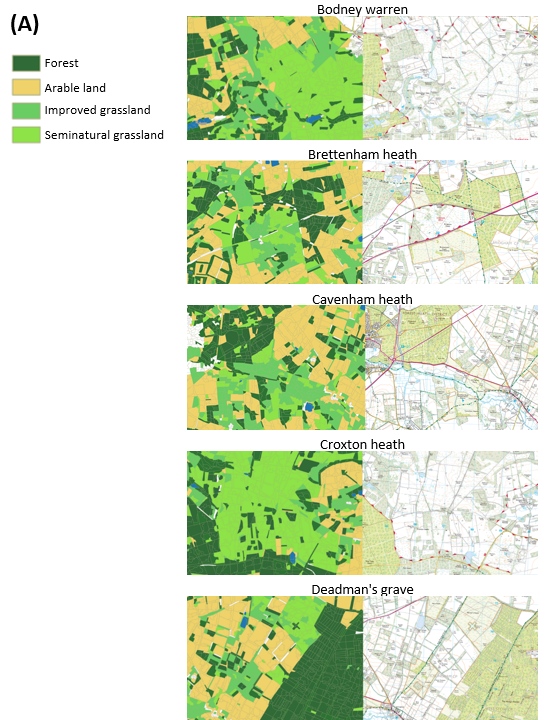


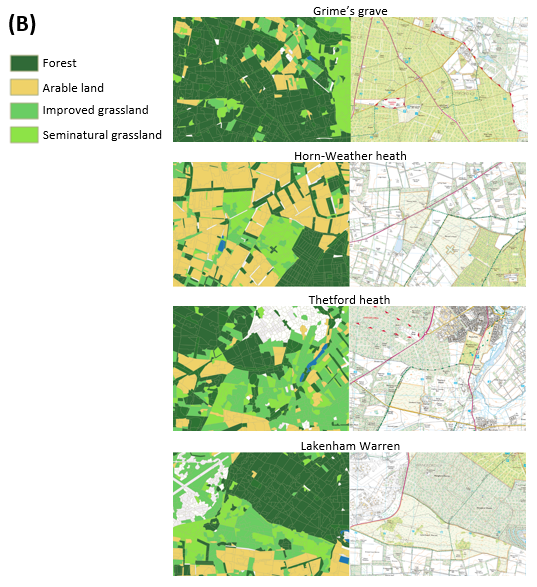


**Fig 1 A and B**: **Grassland misclassification**. Comparison of LCM 2007 land cover classification (left column) and Ordnance survey data (right column, contains OS data © Crown copyright and database right 2019) for a selection of areas surrounding Thetford forest.
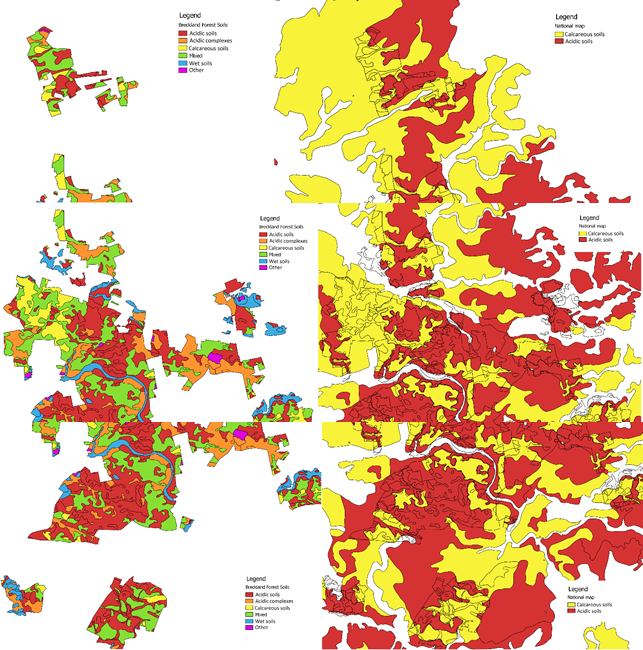


**Fig 2**: Soil map. Mapped examples, relating soil types classified from the Breckland forest soil map (left column) to those mapped by the Cranfield National soil map (right column), see Table S1 for definitions of soils and complexes.

**References**

1. Morton RD, Rowland CS, Wood CM, Meek L, Marston CG, Smith GM. Land Cover Map 2007 (25m raster, GB) v1.2. NERC Environmental Information Data Centre. 2014.

2. Jackson DL. Guidance on the interpretation of the Biodiversity Broad Habitat Classification (terrestrial and freshwater types): Definitions and the relationship with other habitat classifications (]NCC Report No. 307). Peterborough; 2000.

3. Dolman PM, Panter CJ, Mossman HL. The biodiversity audit approach challenges regional priorities and identifies a mismatch in conservation. J Appl Ecol. 2012;49(5):986–97.

4. Corbett WM. Breckland Forest Soils Special Survey 7 [Internet]. Soil Survey of England and Wales. Harpenden, UK; 1973 [cited 2018 Mar 7]. Available from: http://agris.fao.org/agris-search/search.do?recordID=US201303262025

5. National Soil Map of England and Wales - NATMAP [Internet]. Available from: http://www.landis.org.uk/data/nmvector.cfm
