# Supplementary material for: Habitat quality, configuration and context effects on roe deer fecundity across a forested landscape mosaic
Source: PLoS One. 2019 Dec 27;14(12):e0226666. doi: 10.1371/journal.pone.0226666 (PMC6934308; doi:10.1371/journal.pone.0226666)
Supplement: S1 Table — Predicted fecundity of adult roe deer calculated as an unweighted average across raster cells within each of 14 forest subregions from environmental fecundity models and coefficient of variation of forest subregion’s predicted fecundity. (DOCX) [file pone.0226666.s005.docx]

**S5 Table.** **Predicted fecundity per forest subregion**. Predicted fecundity of adult roe deer calculated as an unweighted average across raster cells within each of 14 forest subregions from environmental fecundity models and coefficient of variation of forest subregion’s predicted fecundity.

| **Forest subregion** | **Adult fecundity (n˚embryo per female)** |
| --- | --- |
| Mundford | 1.58 |
| Elveden | 1.52 |
| Mundford West | 1.61 |
| Lynford | 1.55 |
| Croxton | 1.51 |
| Mildenhall | 1.53 |
| Harling | 1.59 |
| West Tofts | 1.52 |
| Didlington | 1.54 |
| Hockham | 1.47 |
| Kings | 1.57 |
| Santon Downham | 1.60 |
| Swaffham | 1.52 |
| High Lodge | 1.51 |
| **CV** | 0.03 |
