# Supplementary material for: Habitat quality, configuration and context effects on roe deer fecundity across a forested landscape mosaic
Source: PLoS One. 2019 Dec 27;14(12):e0226666. doi: 10.1371/journal.pone.0226666 (PMC6934308; doi:10.1371/journal.pone.0226666)
Supplement: Attachment — Submitted filename: Response to reviewers.docx [file pone.0226666.s006.docx]

Dear Franck Jabot

Thank you for returning the reviewers’ comments on our manuscript and for encouraging us to resubmit a greatly modified version of the manuscript. We were pleased that both reviewers were positive about the interest of the paper and made such detailed and constructive comments that have helped us substantially refine the manuscript. We have now substantially revised the manuscript, including making major changes to the analysis, in order to address the reviewers’ comments. We believe the revised manuscript is now substantially improved and clearer and the word length has been reduced.

Below, we copy the original comments of each reviewer (in black font), and beneath each we detail how the manuscript has been amended (in Red).

We would be grateful if you would consider the revised manuscript for publication in PLOS One. Once again, thank you for taking the time to consider our article.

On behalf of all co-authors, yours sincerely

Valentina Zini

**5. Review Comments to the Author**

Please use the space provided to explain your answers to the questions above. You may also include additional comments for the author, including concerns about dual publication, research ethics, or publication ethics. (Please upload your review as an attachment if it exceeds 20,000 characters)

**Reviewer #1:** This paper analyses quite a large data set of hunted roe deer in terms of the landscape drivers of variation in performance as indexed by female body mass and reproductive parameters. The fact that the data are derived from hunted samples imposes some limits on inference (see below), but the analyses and interpretations are nonetheless interesting. The paper is for the most part well-written and quite well referenced (but see below for some notable omissions), although there is some room for further improvements for clarity (see below for some suggestions). There are, however, a number of issues that need to be addressed which I detail below.

We are pleased that the reviewer considers the analyses and interpretations interesting. Below we address the additional detailed comments.

Major Comments:
A. At several points in the manuscript, the authors provide a management-orientated context for their work (e.g. Abstract: lines 10-11). While information on variation in performance in relation to landscape structure can have management implications, there is no such obvious link in the present work. Indeed, data on spatial variation in female fecundity provides only a very limited proxy of population performance because in roe deer, and large herbivores in general, most of the spatial and temporal variation in performance is due to the early survival component of fawns during their first summer, for which the authors have no data. Understanding the link between fecundity and landscape is interesting enough in itself without the need to suggest that there are direct conclusions to inform management from this paper, which is not the case (see detailed comments below)

This was a useful comment, and we have revised the introduction removing the link between cull levels and fertility from the aims. However, while neonatal (as well as yearling and adult) mortality rates are important, knowledge of fertility across the landscape is also necessary for deer management. Since the other reviewer liked our management context, we decided to keep a management framework – partly as this was the motivation to undertake the study. Whether neonatal mortality also varies across a landscape is a separate question, not addressed in this study.

B. A second question that the authors do not adequately address is how they aged animals:
- First, they should provide details on how they aged animals – did they use tooth wear to distinguish yearlings from adults, or did they section teeth to count cementum layers? For the moment they provide no information on this aspect. If the former is the case, aging roe deer from tooth wear is inaccurate. In this paper, the authors distinguish yearling animals (i.e. in their second winter) from older animals (in their third winter) which is somewhat prone to error. Age estimates from tooth sectioning are more reliable. There is not much the authors can do about this, but they should at least state how the animals were aged and also discuss possible error in age estimation.

We are grateful to the reviewer for pointing out this omission. We have now clarified in the methods that age estimation was by tooth eruption, widely used in other studies that distinguishes reliably between juvenile, yearlings and adults.

- Second, the authors use a simple model to justify that they can neglect senescence in this analysis. However, this is not convincing. They have no data on early survival, which is the key parameter, and none on natural mortality of older animals, hence the model has little support. The authors should just say that they could not account for any senescence effects, so that they must assume they are negligible, which is quite likely in a heavily hunted population at low density.

We have clarified in the methods that the Leslie matrix model incorporated study-site specific measures of neonatal survival (obtained from kid:doe field observations) and have therefore retained the Leslie matrix as confirmation that senescence was not confounding in the analysis. We have added that the predicted low % of senescent-aged females is consistent with this being a low density managed (hunted) population, as emphasised by the reviewer. Any additional unmeasured natural mortality of older animals would make the % of ‘senescent-aged’ females even lower than predicted, further supporting our conclusion that it is non-confounding.

C. Third, the authors need to account for the rather poor quality of the spatial information that they have for defining an individual’s range, which is based on shooting location, in the absence of any spatial data.
- First, they need to report what type of hunting was used to sample the population. I presume it is stalking, which would be reassuring as the assumption that the animal is shot within its home range would have more substance (compared to drive hunting which may cause animals to flee prior to death).

This was a useful point, in the revised version of the manuscript we have added that deer were managed by stalking rather than drive-hunting, to support the assumption that deer are shot within their home range (152-153).

- Second, because of the lack of any information on home range, the authors analyse the landscape-performance relationships with a variable size of the buffer to determine what habitat an individual can access. I believe that this approach does not add anything to the message of the paper, is rather confusing, and actually is not biologically convincing. Indeed, the authors suggest that the buffer size that provides the highest explanatory power is over 1000m radius which translates into a range of over 300ha, but this is ecologically not possible. There is a lot of information on the size of home ranges in roe deer, both in this population and elsewhere (e.g. see Morellet et al. 2013 J Anim Ecol for data from across Europe), and they are much much smaller than this buffer, by around a factor of 10, particularly in mixed forest habitat such as that found in the current study. Please choose one biologically-meaningful buffer size and present results of that only – this would simplify everything and be biologically realistic, without detracting from the paper’s message.

This was an important constructive comment, consequently we have substantially revised the analysis in light of this comment to further strengthen the paper. We fully agree with the reviewer that it is appropriate to constrain the buffer sizes according to published information on home range extent. However, we do not have a priori justification for choosing a single buffer size from the ranges presented in the literature. Therefore, we have restricted the buffer sizes to range between 400 and 600m, which correspond to the monthly HR size reported in Morellet et al 2013 J Animal Ecology for Italy, Germany and France and also encompasses roe home range size previously reported for Thetford Forest (Chapman et al. 1993). Using this greatly restricted range (from 400m to 600m, equivalent to 59ha to 113ha), we have retained the MMI approach as an unbiased assessment across this range.

- Third, they need to provide a more informed interpretation of the lack of any significant effects of landscape structure on yearling performance. They report relationships between landscape structure and performance for adults, but did not find evidence for equivalent relationships in yearlings. This could be because of less power due to lower sample size, but it could also be due to the fact that the measures of performance are taken at 20-22 months of age, only shortly after natal dispersal, which occurs at 10-15 months of age in roe deer and concerns around 40% of individuals (Debeffe et al. 2012). As a result, yearling performance is like heavily influenced by the landscape composition of the natal range where around 80% of its body growth took place, which could be very different to the range where the animal was shot. Indeed, did the authors not have body mass data for juveniles? This is likely to be a much better index of performance of the population and the mass should be tightly linked to habitat quality at the shooting site (see Hewison et al. 2009)

We have expanded and clarified our discussion of how natal dispersal (and carry-over effects from the maternal home range) may contribute to the apparent lack of environmental effects for yearlings. We apologise that this was not sufficiently clear in the original version of the manuscript.

D. Effect sizes – it is difficult to get a handle on the effect sizes. The Figures 2 and 3 are not very informative and the reported effect sizes are difficult to interpret. I would like to see the relationships between, for example, mass with arable land and young forest as a figure so we can see the difference in kgs between a very open habitat and an old forested one. A similar type of figure for fecundity would also be informative. As it is, the relationships are not easily accessible.

In the revised manuscript we have now removed the effect sizes from these Figures and have instead presented two new figures showing predictions for supported variables, as requested by the reviewer.

E. By including body mass in the models analysing variation in fecundity, the authors address the question of how landscape structure affects fecundity for a given body mass. While I understand the logic of this, I wonder whether it is the most biologically informed manner to proceed given the well established relationship between mass and fecundity. This merits at least some more discussion. Indeed, the discussion on lines 329-345 is about females being less fecund in more arable areas, BUT it should be discussed that this is for a given body mass, and that you have already shown that body mass is higher in these landscapes! Hence, this probably means that fecundity is almost maximal in arable lands because mass is higher, but that it is not as high as expected given the higher mass. That means that you cannot just interpret this result as fecundity is lower in arable areas (I guess if you remove mass from these models, you will not find the same results). If the authors really wish to determine the relative roles of habitat and mass on fecundity, then path analysis might be appropriate.

This was a particularly helpful comment, that has stimulated us to drill-down further and better clarify the findings at this point. In particular the reviewer considered that “you cannot just interpret this result as fecundity is lower in arable areas (I guess if you remove mass from these models, you will not find the same results)”. In the revised manuscript we have accounted separately and combined for these effects (arable on BM, BM on fertility, arable on fertitliy). This clearly shows, that there is a relatively strong negative effect of arable on fertility, that leads to lower overall fecundity for females with mean BM, and even accounting for the greater BM of roe near the arable boundary, fecundity remains similar or lower at the arable boundary. We have now clarified this in the methods, results and in the discussion, see lines 297-301

F. The paper is quite well-referenced, but there are a few important omissions. Despite the fact that this might appear like self-promotion, the authors have not cited a prominent paper on precisely the same topic in the same biological model: Hewison et al. (2009: Landscape fragmentation influences winter body mass of roe deer. Ecography 32, 1062-1070) analysed the relationship between roe deer body mass and landscape composition in terms of the availability of open habitats (meadows, agricultural fields) and arrived a very similar conclusion, that deer were heavier in more open habitats because of the high quality feeding provided by fertilised crops (values for nutrient levels also supported this conclusion in this work). Hence, the present manuscript provides confirmatory data for this relationship.

We have now cited this paper in the revised discussion

G. In a similar vein, the interpretations based on “source-sink dynamics and dispersal of subordinate animals” is not supported by the existing literature on dispersal ecology of roe deer which has repeatedly shown that dispersers are in better condition than philopatric animals (eg Debeffe et al. 2012).

We are grateful to the reviewer for this useful comment, and have entirely removed this argument from the discussion.

*Detailed comments*

1. Title: It’s a detail but I think the term fecundity is better suited (as most of the variation here is in the number of putative offspring) rather than fertility (which is more usually applied to distinguish reproductive and non-reproductive individuals).

I have replaced the term fertility with fecundity

2. Abstract lines 10-11 and lines 33-34 – As indicated above, the management context of this work is not obvious and is a distraction here. In particular, this paper has nothing to do with source-sink dynamics. In addition, data on spatial variation in female fecundity provides only a very limited proxy of population performance because in roe deer, and large herbivores in general, most of the spatial and temporal variation in performance is due to the early fawn survival component during their first summer, for which the authors have no data.

We have removed some part of the management framework, but not all, see our earlier comment above.

3. Line 14 – not very informative to include these methodological considerations here

We have deleted some of the details

4. Line 32 – this is way too speculative for the abstract. All you can reasonably conclude is that there are unexplained drivers of spatial variation in performance. There is absolutely no support for either density, disturbance or competition effects as you did not have any metrics of these parameters, hence to state this in the abstract is highly misleading.

We have deleted the suggested factors of spatial variation.

5. Lines 40-47 - As described above, the management framework presented here is not appropriate, as the authors do not have any information on whether this system functions as a source-sink, and information on female fecundity is only a very small part of the story. Predicting the cull levels for management aims will not be reliably informed by data solely on fecundity in uteri, as it is recruitment (ie survival to 1 year old) that counts and the most variable parameter is early fawn survival. (Note that the approach using indicators does provide a basis for adjusting cull numbers based on a trial and error approach, see Morellet et al,. 2007). A more straightforward framework for this paper on landscape structure and demographic performance would be more appropriate.

Contrary to the suggestion of the reviewer, we do have evidence suggesting that this forest population acts as a source. The reviewer emphasises a focus on demographic performance, which is what we have done, however we prefer to set this in a landscape-scale management context as this was the motivation for the study. See responses above.

6. Line 69 – there is no direct link between variation in the number of foetuses and the required cull, this should be removed.

Amended accordingly, statement removed.

7. Lines 66-67; the introduction would benefit from a little more justification as to why young stands and certain soil types (this is justified in the Methods, but could better appear in the Introduction), grassland and arable lands are considered high quality for roe deer (as described above, see Hewison et al. 2009). More details on what these terms mean exactly is also required (probably in the Methods section), for example, does grasslands means meadows? And what crops are included in arable? Roe deer are browsers that cannot easily cope with a high cellulose diet and so what is considered a rich habitat varies among seasons (eg see Abbas et al.).

We added these details to the Introduction, see lines 68-73. We have added arable crop types to the methods as well.

8. Lines 100-101: please provide the method used to generate these estimates.

We have clarified that population was estimated using distance sampling thermal imaging

9. Line 106: how was age assessed? (see above)

Done, added tooth eruption as classification method, see above.

10. Lines 114-119: remove and just say that you assume senescent animals to be rare (see above).

We have addressed this comment above.

11. Table 1 – what are the units? And why present this information for each dependant variable data set, it would be better to provide study site averages.

Units (percentage) have been added to Table 1 as requested by the reviewer. Mean and CV are given for each set of modelled buffers, as this illustrates statistical power of the analyses. It is not appropriate to solely quote the mean for the forest, as this would exclude arable.

12. Lines 122-123: please provide more details on what is included in these habitat classes eg crop type.

Added crop type to arable lands.

13. Line 153 - why “particularly over the preceding winter”? You have no spatial information, but in any case roe deer are highly sedentary, and you do not distinguish land types in terms of seasonal availability of resources, so this is not true.

Amended accordingly: the statement “particularly over the preceding winter” has been deleted in the revised manuscript.

14. Lines 157-170: this is unconvincing, please revise as suggested above. First, occasional movements detected by GPS will not have an influence on the landscape-performance relationship as animals will not have access to these resources on a regular basis. Indeed, there is an argument for saying that using only the animal’s core range would provide a better proxy of the resources that it accesses on a daily basis. Second, you have no information on the density dependence of range size, so this is pure speculation. Third, dispersal of roe deer is not density-dependent (see Gaillard et al. 2008) and so this is not appropriate reasoning. Using a biologically realistic buffer with a conservative size (eg 50ha, but definitely no more than 100ha) to include the habitats that are routinely available to an individual would be a much more realistic and biologically informed approach (see above).

We have substantially revised the range of buffer radii used, in accordance with the available empirical evidence in the literature resulting in a maximum buffer of 113ha, as per the earlier comment (above).

15. Lines 175-8 – including forest sub-region and year as random effects is indeed justified to control for spatial/temporal autocorrelation. However, in no way does this account for spatial variation in density or disturbance, or for temporal variation in winter severity – you have absolutely no information on the spatial and temporal structure of these drivers - this is highly misleading at best (see also in abstract).

Including year as a random covariate does control for inter-annual variance in fecundity, including that component which may have arisen due to weather. We agree that forest sub-region is a larger spatial scale than finer-grained variation in recreational access patterns, so will not fully account for this finer scale variation – this is now explicitly acknowledged in the revised methods. Furthermore, in response to the reviewer, we have removed all reference to local deer density and recreational patterns from the abstract (deleting the statement “suggesting factors such as local deer density, inter-specific competition, or recreational disturbance may be important.” that was replaced with “suggesting other factors might be important”).

16. Line 182 – given the low frequency of non-pregnant adults and the possible mis-classification due to late implantation for these animals, it might be worth just removing these individuals.

Late implantation is to a large part controlled for by week as a covariate. We agree that non-pregnant adults are uncommon, but these are a biologically important part of the sample and may be expected to represent biological mechanistic effects. We have therefore retained these in the data.

17. Line 184: as birth is mostly in May and the rut in late July and August, yearlings are impregnated at around 15 months of age.

In the revised manuscript this is now corrected to 15 months of age

18. Lines 201-3: Auto-correlation among habitat variables: while I understand the difficulty of dealing with landscapes in which the combination of available habitats necessarily sums to 1, I do wonder whether the strong relationship (up to 25% of the variation in one is explained by the other) between the availability of arable lands and young forest is a problem for interpretation. Using a more realistic buffer size (see above) would appear to minimise this problem.

The reviewer was correct and we did indeed find that using a narrower, and biologically justified, range of buffer radii removed this inter-correlation between arable and young forest. This allowed us to substantially simplify analysis and presentation of the results, without the need for two sets of models. We also note that the modelled land cover variables do not sum to one, due to varying amounts of predominant pole stage that is not included as a predictor in models.

19. Line 303: I think you should leave it to the reader to evaluate the power of the study, please re-phrase.

Revised accordingly, removing the word ‘powerful’

20. Line 304 – 16 or 14 years, be consistent.

Now corrected

21. Lines 306-15: here you seem almost to be telling us how large the home ranges are based on your analysis. This is clearly not the case, and home ranges of this size are very very unlikely (see above). Please present a single analysis based on a realistic buffer size for roe deer.

We have now substantially revised the analyses, using a restricted range of buffer size (400 to 600m)

22. Lines 320 – what types of crops?

Now specified in methods, see responses above

23. Lines 346 – 57: this is more likely due to the intervention of natal dispersal between body mass growth and sampling for reproductive performance (see above). The discussion of adults moving their home range is highly speculative and not supported by the roe deer movement ecology literature which repeatedly has shown that adults are highly sedentary. The pers comm. reference to one of the authors is highly unusual to say the least, and not convincing (based on unmarked animals?), as adult roe deer almost never move such distances outside of migratory events, whereas natal dispersal does include movements of such length at around 12 months of age.

We have now removed the speculative text considering adult movement from the revised discussion

24. Lines 371-4: this interpretation is not supported as the relationship between fecundity and the cull required is, at very best, indirect (see above).

The discussion of management implications has been removed, in response to earlier comments (see above)

Mark Hewison

**Reviewer #2**: Comments to the authors
The MS investigates the the effects of local variation in habitat quality on fertility and body mass in roe deer across a forested landscape mosaic. The study based on a large sample of individuals culled during 2002-2015 provides a methodological approach useful in the wildlife management of the species in large areas.

We are pleased the reviewer considers this study to make a useful contribution

*Specific comments:*
• line 101. In order to evaluate interspecific competition, did you extimate the consistence of red deer, of fallow and of muntjac in the study sites? Please comment in M. and M.

We haven’t explicitly evaluated any effects of interspecific competition, as our variables were habitat and landscape variables.

• In M and M you considered environmental variables analysing two model sets: 1) arable lands, grasslands, and calcareous soil; 2) young forest, grasslands, and calcareous soil. Nevertheless, it would have been useful to specify: a) in arable lands, crop cultures and their incidence; b) floristic composition and, if not possible, dominant species composing vegetation in candidate environmental variables.
In the revised manuscript, we have now specified crop types in the methods (see also response to Reviewer #1 who made a similar request). It is not practicable to list dominant plant species as vegetation is diverse (on differing soil types).

• Line 140: (30,31). Please add also the following citation:
Freschi P, Fascetti S, Riga F, Cosentino C, Rizzardini G, Musto M (2017) Diet composition of the Italian roe deer (Capreolus capreolus italicus) (Mammalia: Cervidae) from two protected areas. Eur Zool J 84(1):34–42. [https://doi.org/10.1080/11250003.2016. 1268655](https://doi.org/10.1080/11250003.2016.%201268655)

Added accordingly

• Lines 306-309: “were much larger than home range extents previously found by telemetry studies”. In your opinion, could hunting activity or competition with other species be the cause of a so large home range? Please comment.

We have restricted the range of buffers modelled to the range appropriate for HRs found for roe deer in Morellet et al (2013) and Chapman et al (1993), to 400 to 600m, see above. As we are not estimating the functional Home Range of the study animals this section of discussion has now been removed from the revised manuscript.

• Line 317: Please add also the following citation:
◦ Freschi P, Fascetti S, Riga F, Cosentino C, Rizzardini G, Musto M (2017) Diet composition of the Italian roe deer (Capreolus capreolus italicus) (Mammalia: Cervidae) from two protected areas. Eur Zool J 84(1):34–42. https://doi.org/10.1080/11250003.2016. 1268655
We added the reference accordingly

• Line 323: ” Contrary to our predictions, no effect of calcareous soil was found on roe body mass.” “Calcareous soil” or vegetation grazed on calcareous soil? Please specify.

We assume it is the vegetation found on calcareous soil; this is now explicit in the abstract, and introduction.

• The authors should highlight in “discusssions” the importance of feeding preferences of the species in the studied sites. These items, widely utilised in medium and in larger herbivores, are stategic in wildlife managements. Please refer to the following articles:
◦ Bugalho, M. N., & Milne, J. A. (2003). The composition of the diet of red deer (Cervus elaphus) in a Mediterranean environment: a case of summer nutritional constraint?. Forest Ecology and Management, 181(1-2), 23-29.
◦ Freschi P, Fascetti S, Musto M, Mallia E, Blasi AC, Cosentino C, Paolino R (2014) Diet of the Apennine hare in a Southern Italy Regional Park. Eur J Wildl Res 60(3):423–430. https://doi.org/10.1007/ s10344-014-0799-y
◦ Freschi P, Fascetti S, Musto M, Mallia E, Cosentino C, Paolino R (2015) Diet of the Italian hare (Lepus corsicanus) in a semi-natural land- scape of southern Italy. Mammalia 79(1):51–59. https://doi.org/10. 1515/mammalia-2013-0117.
◦ Freschi, P., Fascetti, S., Riga, F., Cosentino, C., Rizzardini, G., & Musto, M. (2017). Diet composition of the Italian roe deer (Capreolus capreolus italicus)(Mammalia: Cervidae) from two protected areas. The European Zoological Journal, 84(1), 34-42.
◦ Krasnov, V., Shelest, Z., Boiko, S., Gulik, I., & Sieniawski, W. (2015). The diet of the roe deer (Capreolus capreolus) in the forest ecosystems of Zhytomirske Polesie of the Ukraine. Forest Research Papers, 76(2), 184-190.
◦ Nagarkoti, A., & Thapa, T. (2007). Food habits of barking deer (Muntiacus muntjac) in the middle hills of Nepal. Hystrix, the Italian Journal of Mammalogy, 18(1).
◦ Puig, S., Videla, F., Cona, M. I., & Monge, S. A. (2007). Diet of the brown hare (Lepus europaeus) and food availability in northern Patagonia (Mendoza, Argentina). Mammalian Biology-Zeitschrift für Säugetierkunde, 72(4), 240-250.

We did not find it useful to expand the discussion of dietary preferences beyond the existing statements, and have not expanded to consider diet of other herbivores not considered in the study.
