# Supplementary material for: Habitat quality, configuration and context effects on roe deer fecundity across a forested landscape mosaic
Source: PLoS One. 2019 Dec 27;14(12):e0226666. doi: 10.1371/journal.pone.0226666 (PMC6934308; doi:10.1371/journal.pone.0226666)
Supplement: Attachment — Submitted filename: Response to reviewers.docx [file pone.0226666.s007.docx]

Below, we copy the original comments of each reviewer (in black font), and beneath each we detail how the manuscript has been amended (in Red).

**Review Comments to the Author**

Please use the space provided to explain your answers to the questions above. You may also include additional comments for the author, including concerns about dual publication, research ethics, or publication ethics. (Please upload your review as an attachment if it exceeds 20,000 characters)

Reviewer #1: Dear authors

Your manuscript deals with a interesting study on the effect of Habitat quality on roe deer fecundity across a forested landscape mosaic. Suggestions were exhaustively accepted and now the paper is improved and well written.

Reviewer #2: General comments
This paper analyses quite a large data set of hunted roe deer in terms of the landscape drivers of variation in performance as indexed by female body mass and reproductive parameters. This is a revised version of a manuscript that I reviewed some time ago. The authors have made considerable effort in revising the paper in relation to my comments and those of a second referee. I found that the revised version was significantly improved overall, although there are some issues that I highlighted that the authors have preferred not to account for. Because some of these issues could be seen as related to personal viewpoint, at the end of the day this is a matter of choice and does not preclude publication. I have tried to explain why I do not necessarily agree with some of these choices below, but I leave it up to the authors to judge. However, there is one major point that requires further clarification. I still cannot see an analysis of the raw data relationship between female fecundity and the proportion of arable land irrespective of body mass, i.e. I want to see an analysis (and figure) of this relationship when body mass is not included in the model. Otherwise, I have difficulty buying the interpretation that a negative relationship between fecundity and % arable land offsets the advantage obtained through higher body mass in more arable areas (see below).

This was a very good point and we have now added a new graph showing prediction from a model that ignores body mass completely to show that there is infact a negative relationship between arable and fecundity despite the positive relationship between arable and body mass.

Major Comment:
A. From the new figures, it is somewhat easier to get a handle on the relationships. Indeed, it is clear from the Figure 3 that the effect of arable land on body mass is quite strong and robust. In contrast, it is also clear that there is very little support for any biologically meaningful relationship between % arable land and fecundity, while controlling for body mass (Fig. 3C). Indeed, firstly, the confidence intervals for estimates of fecundity at 5% and 80% arable land clearly overlap. Hence, I do not buy the interpretation on lines 296-301 that fecundity decreases with increasing proportion of arable land. Secondly, I still do not see the equivalent analysis of the raw data relationship between fecundity and % arable land ie IGNORING body mass completely in the model. Given the strong relationship between fecundity and body mass that they show, I expect that the relationship between fecundity and % arable is also positive if you remove body mass from the fecundity model altogether. Please show this. Otherwise the interpretation that the body mass advantage of having access to arable land is offset by a negative effect on fecundity is not convincing.

We have now added a new graph showing prediction from a model that ignores body mass completely

Other comments
B. Concerning the management-orientated context for this work, of course I agree that information on variation in female fecundity in relation to landscape structure could have management implications. However, in almost all contexts where ungulate population dynamics have been studied to date, variation in this component is swamped by spatial and temporal variation in the early survival component. As a result, population performance is more informatively indexed by fawn survival during their first summer. Therefore I would question whether indeed “knowledge of fertility across the landscape is also necessary for deer management”. However, I recognise that this is, to a certain degree, a question of personal opinion.

C. While the authors have now provided details on how they aged the animals, despite what they say on line 107, I know of no study that has demonstrated that tooth eruption provides reliable information to distinguish yearling animals in roe deer. What is the basis for this assumption? The error is not huge for yearlings (e.g. see Hewison et al. 1999), but it should at least be acknowledged.

We have now acknowledged the error and cited the study.

D. For the senescence aspect, you can make this assumption if you wish, and intuitively I expect that it is a fair assumption given the hunting pressure, but I strongly dispute that you have reliable estimates of observed early survival as you can only get this information if you mark fawns at birth. Female:fawn ratios are an unreliable proxy for this in roe deer, as it is virtually impossible to distinguish yearlings (ie non-reproductive) from adult females in autumn. Hence, the observed ratio is strongly dependent on population age structure, e.g. if performance is high, then there will be proportionately more yearlings in the population, which will artificially decrease the observed female:fawn ratio because yearlings (18 months old in autumn) have not yet reproduced. This means that a high performance population may have a lower female:fawn ratio simply because of the fact that there are more immature females in the population which are impossible to reliably distinguish and so are included in the calculation of the ratio. As I previously said, the authors should just say that they could not account for any senescence effects, so that they must assume they are negligible, which is quite likely in a heavily hunted population at low density.

We have now deleted the Leslie matrix and said we couldn’t account for senescence effects.

E. In terms of the putative source-sink dynamics of this system, I remain unconvinced. Source-sink dynamics cannot be inferred from hunting data alone. A source sink system implies that the sink population is unable to sustain itself (r < 1) in the absence of immigration from the source population (r > 1). Just because there is variation in performance between neighbouring populations, one cannot therefore assume that the system contains a source and sink without detailed information on i/ population growth rate AND ii/ net movement of animals between the two sites. I do not see how the authors can obtain such information on movements from hunting data of non-marked animals. I therefore strongly suggest that this consideration be removed, e.g. first sentence of abstract and elsewhere.

We agree that fertility is only a small part of the whole source sink-system and that other, more important informations about the system are required to infer sources and sinks. Our intention with the paper is solely to address how fertility changes throughout the landscape and whether it is or it isn’t an important factor to account for, when looking at sources and sinks (provided all the other informations are used to build sources and sinks, such as density of deer, mortality rate and juvenile survival).

Mark Hewison
